# Mutations found in cancer patients compromise DNA binding of the winged helix protein STK19

**DOI:** 10.1038/s41598-024-64840-9

**Published:** 2024-06-18

**Authors:** Jian Li, Xinli Ma, Xiaoyu Wang, Xiaotong Hu, Shaobo Fang, Guoguo Jin, Kangdong Liu, Zigang Dong

**Affiliations:** 1 China-US (Henan) Hormel Cancer Institute, Zhengzhou, 450003 Henan China; 2 Department of Pathophysiology, School of Basic Medical Sciences, Zhengzhou University, Zhengzhou, 450001 Henan China; 3 Henan Key Laboratory of Chronic Disease Management, Fuwai Central China Cardiovascular Hospital, Zhengzhou, 450000 Henan China

**Keywords:** Biochemistry, Structural biology

## Abstract

Serine/threonine protein kinase 19 (STK19) has been reported to phosphorylate and activate oncogenic NRAS to promote melanomagenesis. However, concerns have been raised about whether STK19 is a kinase. STK19 has also been identified as a putative factor involved in the transcription-coupled nucleotide excision repair (TC-NER) pathway. In this study, we determined the 1.32 Å crystal structure of human STK19. The structure reveals that STK19 is a winged helix (WH) protein consisting of three tandem WH domains. STK19 binds more strongly to double-stranded DNA and RNA (dsDNA/dsRNA) than to ssDNA. A positively charged patch centered on helix WH3-H1 contributes to dsDNA binding, which is unusual because the WH domain typically uses helix H3 as the recognition helix. Importantly, mutations of the conserved residues in the basic patch, K186N, R200W, and R215W, are found in cancer patients, and these mutations compromise STK19 DNA binding. Other mutations have been predicted to produce a similar effect, including two mutations that disrupt the nuclear localization signal (NLS) motif. These mutations may indirectly impact the DNA binding capacity of STK19 by interfering with its nuclear localization.

## Introduction

The nucleotide excision repair (NER) pathway is the predominant mechanism by which bulky adduct DNA damage is repaired. The NER pathway can be divided into four essential steps: recognition/verification, incision/excision, resynthesis, and ligation. Depending on the damage recognition step, there are two sub-pathways, global genome nucleotide excision repair (GG-NER) and TC-NER. The two sub-pathways later converge at the lesion verification step. In TC-NER, upon DNA lesions RNA PolII stalls, and lesions were detected by the Cockayne syndrome proteins CSA and CSB. Xeroderma pigmentosum (XP) complementation group proteins (XPA-XPG) function in various steps of NER, and mutations in *XPA-XPG* are associated with an increased risk of skin cancer and various internal tumors^1,2^.

The *STK19* gene (alternative name *G11*) was originally thought to encode a nuclear serine/threonine protein kinase that phosphorylates CSN1S1/alpha-casein^3^. Years later, large-scale genomic analysis identified recurrent mutations in the *STK19* gene (D89N, isoform G11Z or G11-Z-short) and suggested that *STK19* may be a driver gene for melanoma and cutaneous basal cell carcinoma^4,5^. STK19 was recently found to phosphorylate and activate oncogenic NRAS to promote melanomagenesis, and the D89N mutant interacts better with NRAS^6,7^. Currently, STK19 is listed as an atypical kinase in several influential databases, including Kinase.com^8^, and the Kinome Atlas^9^. Surprisingly, concerns have been raised about whether STK19 is a kinase. At the protein level, G11Z or G11-Z-short isoforms are not expressed in melanocyte cell lines and two commonly used human cell lines (HEK293 and HeLa). Thus, the "cancer-driving" STK19 D89N substitution is located in the promoter region. Furthermore, STK19 is exclusively nuclear and chromatin-associated, could not be convincingly aligned to any known protein kinase using the Phyre structure prediction tool, and no kinase activity has been detected by another group^10^. Therefore, the function of STK19 as a kinase is highly controversial.

Meanwhile, another study showed that STK19 interacts with CSB after DNA damage, is recruited to the DNA damage site, and its knockdown inhibits transcription recovery after UV irradiation^11^. Furthermore, two independent CRISPR screens identified the *STK19* gene as required for Illudin S tolerance^12,13^. Illudin S induced DNA damage is ignored by global repair and exclusively processed by transcription- and replication-coupled repair pathways^14^. Collectively, these studies strongly suggest that STK19 can be assigned to the transcription-coupled nucleotide excision repair (TC-NER) pathway, but the role of STK19 in TC-NER is poorly understood^2,12^. Interestingly, similar to the *XPA-XPG* genes, alterations in *STK19* have been linked to melanoma^4^, cutaneous basal cell carcinoma^5^, prostate cancer^15^, and aerodigestive squamous cell cancers^16^.

In this study, we determined a 1.32 Å crystal structure of human STK19 protein, revealed that STK19 is a winged-helix (WH) protein instead of a kinase, and performed structural-guided analysis of the STK19 molecular function. In contrast to the conclusions from a recently published STK19 structure study^17^, we provide evidence that STK19 is a monomer in solution, and demonstrate mutations found in cancer patients, K186N, R200W, and R215W, significantly compromise STK19 DNA binding, with more mutations predicted to do so. Additionally, we point out that the DNA binding surface identified so far is different from the canonical nucleic acid binding site of the WH domain.

## Results

### Structural analysis of human STK19 reveals a winged helix protein

Our analysis focused on the human *STK19* isoform G11-Y-short (UniProt accession number P49842-4, 254 amino acids), which was designated as the canonical sequence. This isoform lacks the 110-amino acid N-terminal portion of isoforms G11Z and G11-Z-short. The existence of the latter two isoforms is controversial^6,7,10^. We determined the 1.32 Å crystal structure of human STK19, containing residues 31-254. This fragment lacks the predicted nuclear localization signal (NLS) only at the N-terminus of the full-length protein^18^, so we refer to the crystallized fragment as STK19 in the following sections.

**Figure 1:**
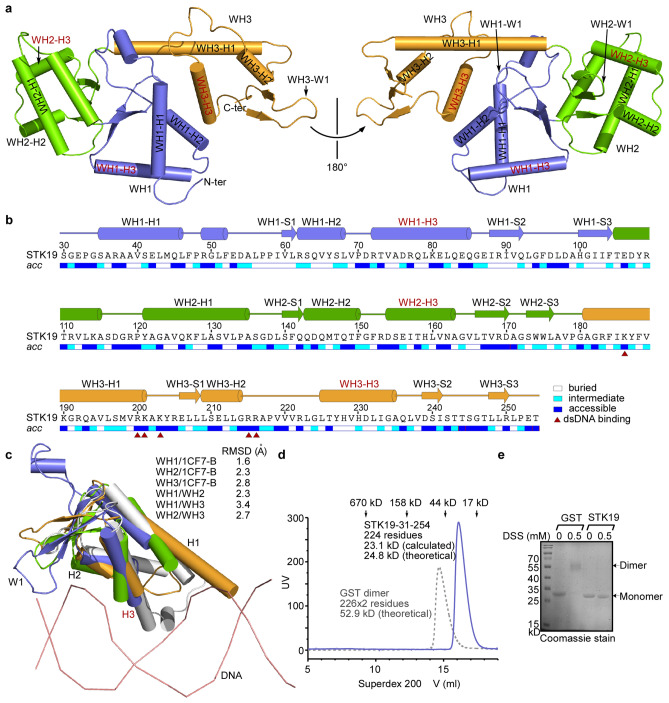
Overall structure of the human STK19 protein. (**a**) Structure of the STK19 reveals a compact fold consisting of three winged-helix (WH) domains (WH1-WH3) packed together. The three WH domains are colored blue, green, and orange, respectively. Helix 3 (H3) is known as the recognition helix. (**b**) Amino acid sequence, solvent accessibility (*acc*), secondary structure, and domain organization of the crystalized STK19 fragment. Key residues later found crucial for dsDNA binding are labeled with red triangles. The solvent accessibility bar was produced by ENDscript^38^. Residue S30 is derived from the expression vector, and the sequence harbors C116S/C136S/C240S mutation (see methods). (**c**) The STK19 WH domains share a similar fold with a representative WH domain in the structure of transcription factor E2F-DP bound to their cognate DNA (PDB ID 1CF7, chain B). Note the helices H3 contact the major groove of the modeled DNA.The root-mean-square deviation (RMSD) of aligned C-alpha atoms between these domains are listed. (**d**) The STK19 protein is a monomer in solution. The molecular weight of 23.1 kD was calculated for the STK19 based on the gel filtration elution volume, which is well-separated from the dimeric Glutathione S-transferase (GST) protein having almost the same number of residues. (**e**) Crosslinking by Disuccinimidyl suberate (DSS) followed by SDS-PAGE analysis indicates that STK19 is a monomer in solution, in contrast to the GST protein.

The structure of STK19 reveals a compact fold consisting of three winged helix (WH) domains (WH1-WH3) packed together (Fig. 1a and b). This was confirmed by comparing the STK19 structure against those in the Protein Data Bank (PDB) with the DALI server^19^. In the absence of a kinase domain, the current official protein and gene names of STK19 need to be changed to better reflect its identity. The same conclusion was achieved by another two independent studies^10,17^.

Classically, the WH domain comprises three α-helices (H1-H3), three β-strands (S1-S3), and two characteristic loops (the so-called ‘wings’, W1-W2). These secondary structure elements are arranged in the order H1-S1-H2-H3-S2-W1-S3-W2 (Fig. 1b). The WH domain is a widespread nucleic acid binding fold, with helix H3 being the recognition helix and W1 frequently involved^20^. The three WH domains of STK19 share a similar overall fold with a representative WH domain in the structure of the transcription factor E2F-DP bound to its cognate DNA^21^. As expected, helix H3 contacts the major groove of the modeled DNA (Fig. 1c). Notably, the WH3 nucleic acid recognition helix (WH3-H3) is less accessible compared to WH1-H3 and WH2-H3, owing to its proximity to WH1-H2 (Fig. 1a and Supplementary Fig. S1a).

Alignment of the STK19 structure from this study and the recently published STK19 structure reveals rigid and flexible parts of the protein. The spatial arrangement of the three tandem WH domains is quite stable. Meanwhile three loops, the WH1-W1, WH3-W1, and the loop between H1-S1 in the WH1 domain exhibit large structural variations (Supplementary Fig. S1b). The STK19 protein in solution is monomeric, as suggested by analytical gel filtration, run in parallel with dimeric Glutathione S-transferase (GST) protein of similar size and with gel filtration standards (Fig. 1d). This conclusion is also supported by chemical crosslinking using Disuccinimidyl Suberate (DSS). In this experiment, a dimeric band could be readily detected for GST protein in SDS-PAGE, while under the same condition, STK19 protein is predominantly a monomer (Fig. 1e). This is different from the conclusion of the recent paper^17^. The latter emphasized more about the capability of STK19 to form dimers, especially in the presence of DNA, which is still possible.

### STK19 binds more strongly to double-stranded DNA and RNA (dsDNA/dsRNA)

**Figure 2:**
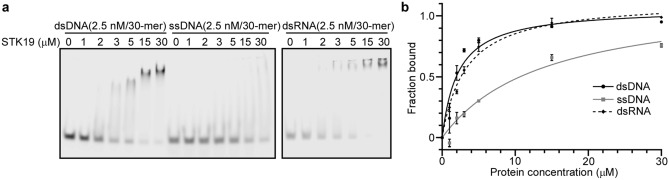
STK19 binds more strongly to double-stranded DNA and RNA. (**a**) Binding of the STK19 protein to dsDNA (30-mer (dT-dA)30), ssDNA (30-mer dT30), and dsRNA (30-mer (rU-rA)30). Representative images of EMSA are shown. (**b**) Quantification of (A) from multiple independent repeats. The dissociation constants estimated at the half-maximal binding are 2.2, 14.1, and 3.2 µM for the dsDNA, ssDNA, and dsRNA, respectively. n = 4 for dsDNA and ssDNA measurement. n = 2 for dsRNA measurement. Error bars represent error of the mean (SEM).

Because the WH domain is well-known for its nucleic acid binding capability, we tested whether STK19 has this function. Electrophoretic mobility shift assay (EMSA) results showed that STK19 binds dsDNA (30-mer (dT-dA)30) and dsRNA (30-mer (rU-rA)30), while its binding to ssDNA (30-mer dT30) is diminished (Fig. 2a). Assuming one site-specific binding model, the dissociation constants estimated at the half-maximal binding are 2.2, 14.1, and 3.2 µM for the dsDNA, ssDNA, and dsRNA, respectively (Fig. 2b). Thus, STK19 displays preferred binding to double-stranded nucleic acid.

### A basic patch centered on the helix WH3-H1 contributes to dsDNA binding

**Figure 3:**
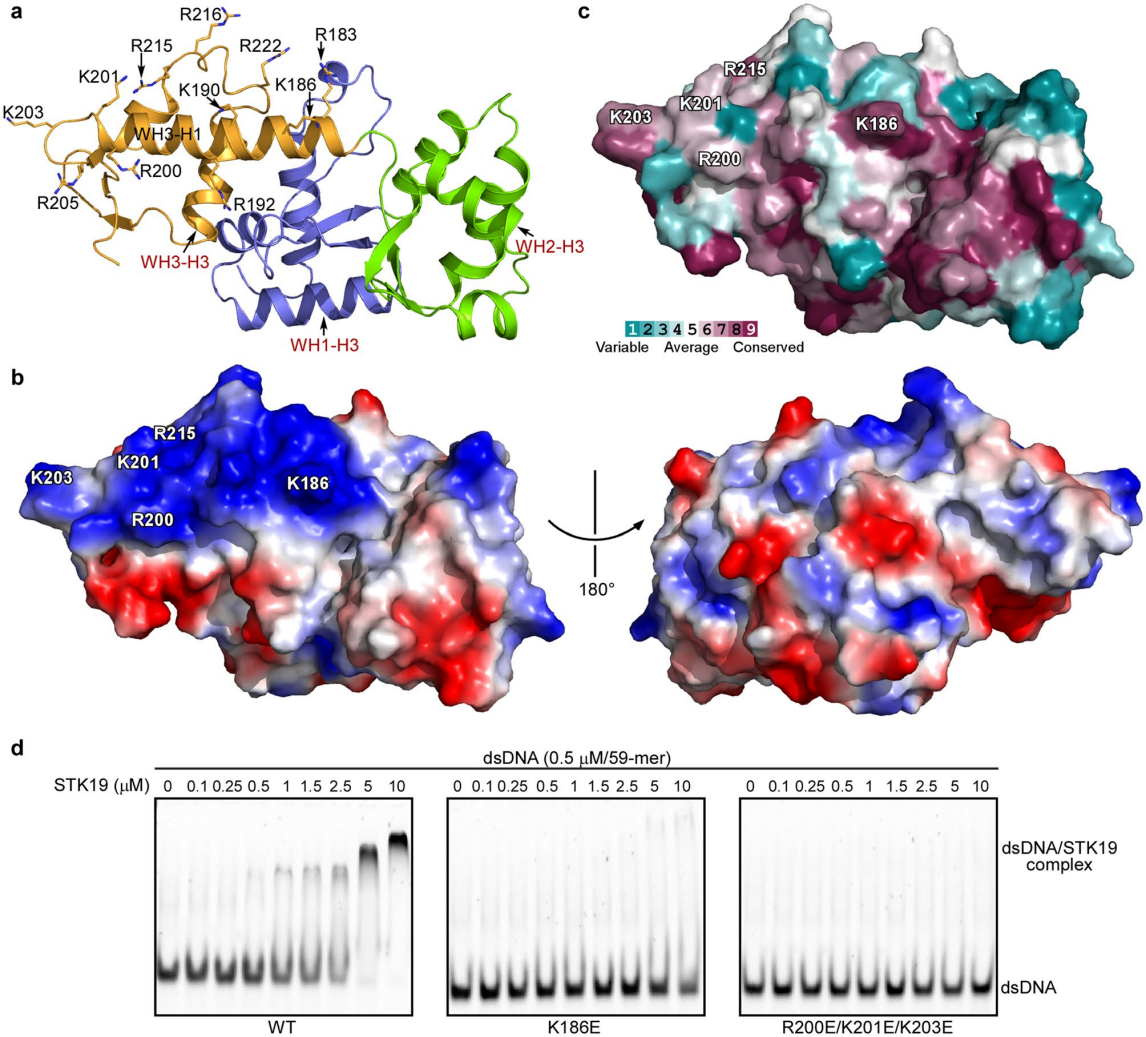
A basic patch centered on the helix WH3-H1 contributes to dsDNA binding. (**a**, **b**) Electrostatic surface analysis of the STK19 structure reveals a large patch of basic residues centered around the WH3-H1 helix. The involved residues are labeled. (**c**) Surface representation showing the distribution of conserved residues from ConSurf analysis^23^ with default settings (conservation among 150 homologous STK19 sequences). The relatively conserved basic residues are labeled. (**a**) and (**c**) are of the same view. (**d**) K186E and R200E/K201E/K203E mutations disrupt the STK19 DNA binding ability. Representative images of EMSA are shown.

Protein-DNA interfaces tend to be enriched with positively charged residues^22^. Electrostatic surface analysis of the STK19 structure reveals a large patch of basic residues centered on the WH3-H1 helix. These residues include R183, K186, K190, R192, R200, and K201 of the helix WH3-H1, K203 and R205 in the following loop, and R215, R216, and R222 in the H2-H3 loop of the WH3 domain (Fig. 3a and b). Conserved surface residues represent functionally critical regions of a protein. Through Consurf server analysis^23^, it was found that residues K186, R200, K201, K203, and R215 are relatively conserved, among these basic patch residues (Fig. 3c). Mutations of these conserved residues, namely K186E, R200E/K201E/K203E, and R215E/R216E, abolished dsDNA binding (Fig. 3d and Supplementary Fig. S2a). Importantly, conserved residues from the classical nucleic acid recognition helices (helix H3) of the three WH domains do not contribute to dsDNA binding in our EMSA assay (Supplementary Figs. S2b and S3). In summary, a basic patch with conserved residues K186, R200, K201, K203, and R215 contributes to dsDNA binding.

### Mutations found in cancer patients compromise STK19 dsDNA binding

**Figure 4:**
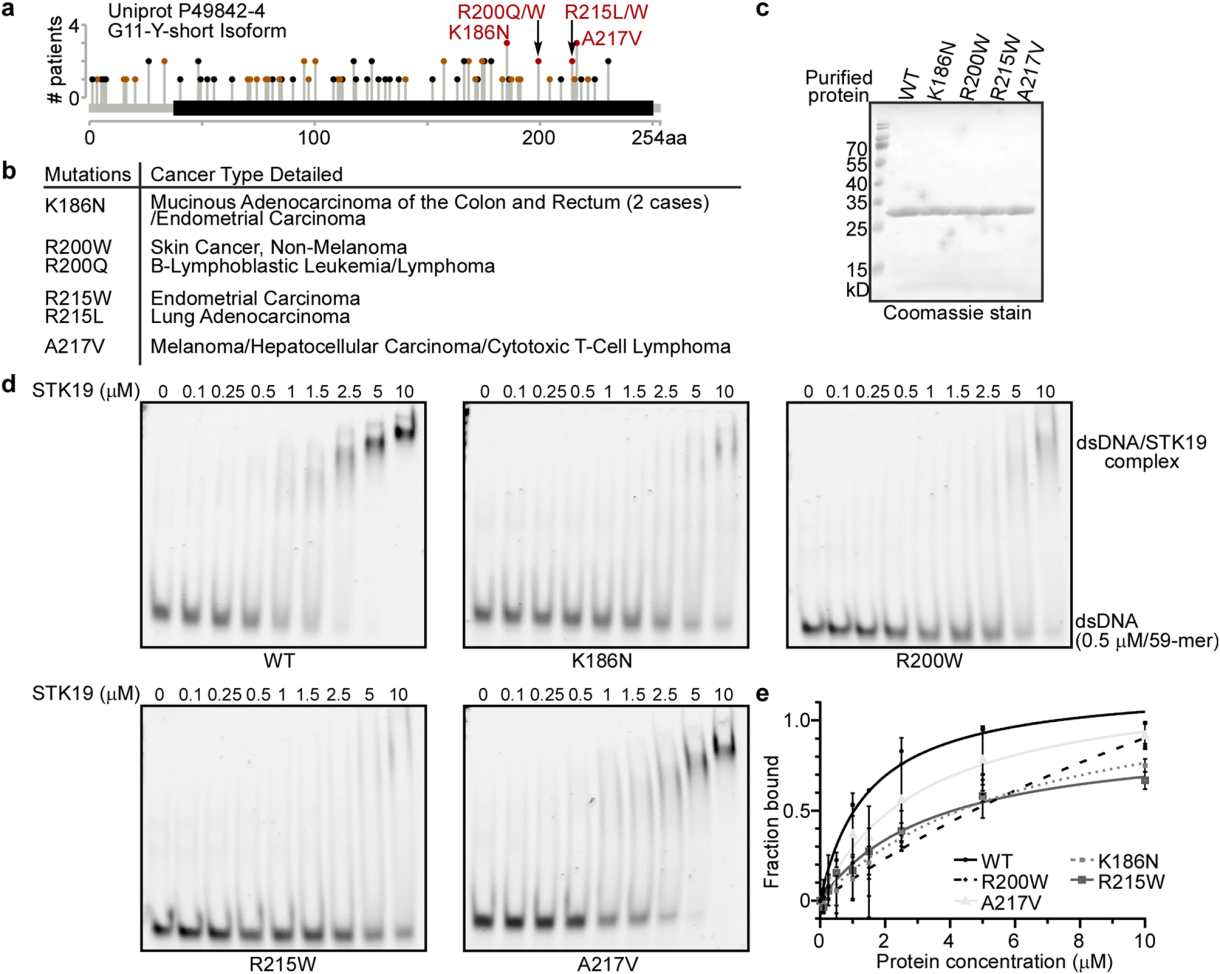
Mutations found in cancer patients compromise STK19 DNA binding. (**a**) Distribution of mutations found in cancer patients (cBioPortal curated set of non-redundant cancer studies) along the STK19 protein. Mutations tested in this study are colored red, while mutations that are predicted to interfere with the dsDNA binding function of the STK19 (see discussion) are colored orange. (**b**) Cancer types associated with the selected patient mutations. (**c**) Purified proteins used in the EMSA assay. (**d**) Mutations K186N, R200W, and R215W significantly compromise STK19 dsDNA binding ability. Representative images of EMSA are shown. (**e**) Quantification of (**d**) from independent repeats. n = 2. Error bars represent SEM.

Mutations of the XP group genes in the NER pathway are genetic predispositions to cancer. This sparked our interest in STK19. Surprisingly, mutations found in cancer patients (cBioPortal curated set of non-redundant cancer studies^24,25^) involve the DNA binding residues identified above, among which K186N appeared in three cases (Fig. 4a and b). K186 is also the most conserved basic residues being studied (Fig. 3c). No particular type of cancer shows higher co-occurrence with STK19 mutation (Fig. 4b), and the *XPC*, *XPE*, and *STK19* genes have similar somatic mutation frequencies. We selected and purified to homogeneity four STK19 protein variants harboring the patient mutation (Fig. 4c). Of the investigated mutations, K186N, R200W, and R215W significantly compromised STK19 dsDNA binding ability (Fig. 4d and e).

## Discussion

**Figure 5:**
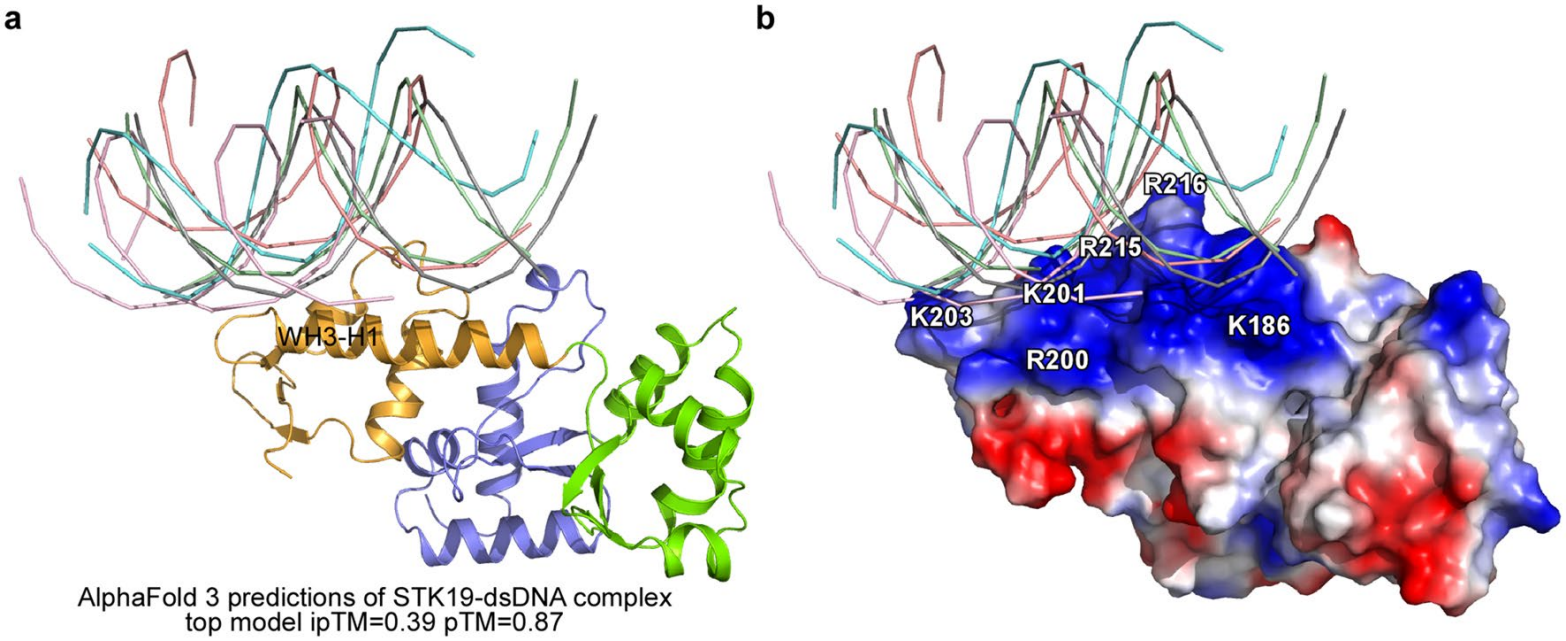
AlphaFold 3 model of the STK19-dsDNA complex. (**a**) The STK19-dsDNA binding sites are centered on the WH3-H1 helix in all five predictions given by the AlphaFold 3 server. These models have a pTM ranging from 0.87 to 0.86, and ipTM between 0.39 and 0.36. A 15-mer (dT-dA)15 DNA was used as input, and trajectories of dsDNA in all five models are shown. (**b**) Same as in (**a**), with the electrostatic surface displayed and highlighting the charged residues involved in DNA binding. The modeling is in agreement with our experimental evidence.

In this study, we show that a basic patch centered around the helix WH3-H1 mediates dsDNA binding (Fig. 3). However, this is not the canonical nucleic acid binding mode of the WH domain, which normally uses the H3 recognition helix and the W1 wing^20^. To complement this finding, we utilized the latest AlphaFold 3 server to predict a model of the STK19-dsDNA complex structure^26^. The input dsDNA was a 15-mer (dT-dA)15, which is half the length of the dsDNA used in Fig. 2 to measure the binding affinity. Gratifyingly, the dsDNA occupies the WH3-H1 basic patch in all five predictions, although the dsDNA trajectories are slightly different. Overall, the modeling is consistent with our experimental evidence (Fig. 5).

Trying to provide a more in-depth insight, we split the STK19 structure into each of the three WH domains (WH1-WH3), and searched for similar structures bound with DNA using the DALI server. The STK19 WH1 exhibits high similarity with the structure of riboflavin kinase in complex with its cognate DNA operator (PDB ID 5TRD, chain A, DALI Z-score 6.8, RMSD 2.3). The aligned region maps to the WH domain but not the riboflavin kinase domain (CTP-dependent) of this flavokinase. Structural superimposition shows that the STK19 WH1 might be compatible with dsDNA binding. Two extremely conserved residues, T73 and D76 of the STK19 WH1-H3 helix could mediate interactions with the DNA bases, which is often seen in the WH domains recognizing specific sequences (Supplementary Fig. S3a–c). The STK19 WH2 resembles the structure of the zinc uptake regulator complexed with the cognate promoter (PDB ID 4MTD, chain D, DALI Z-score 6.7, RMSD 2.6). Similarly, two highly conserved residues, D154 and T158 of the WH2-H3 helix are at positions close to the superimposed dsDNA bases (Supplementary Fig. S3d–f). A top hit for the STK19 WH3 domain is the structure of the viral Zalpha domain bound to left-handed Z-DNA (PDB ID 1SFU, chain A, DALI Z-score 6.7, RMSD 3.4). The Z domain is a special form of the WH domain that recognizes and stabilizes the Z-DNA (or Z-RNA conformation)^20^. Structure alignment shows that the STK19 WH3 may not be optimal for canonical dsDNA binding, due to steric clashes between the adjacent WH1 domain and the modeled DNA. Furthermore, the predicted DNA binding surface is not as conserved as that of the WH1 and WH2 domains (Supplementary Fig. S3g–i). Despite those possibilities, when tested in EMSA assays, the aforementioned conserved residues on the recognition helices do not contribute to dsDNA binding (Supplementary Fig. S2b). Taken together, the above analysis again shows that our proposed STK19 dsDNA binding model (Fig. 5) is quite different from models of the classical WH domains. In the current study, only arbitrarily chosen and random DNA sequences were used. Given the fact that WH domains are frequently involved in sequence-specific, structure-specific^20,27,28^, and modification-specific^29,30^ nucleic acid binding, whether STK19 protein has similar properties, especially in cells under a physiological setting, such as in the NER pathway dealing with bulky adduct DNA damage, requires further in-depth research. Lastly, STK19 could also participate in protein–protein interactions^20^.

Importantly, we demonstrate mutations found in cancer patients, K186N, R200W, and R215W, significantly compromise STK19 DNA binding (Fig. 4). Apart from the experimentally validated DNA binding mutants (Fig. 4a, colored red), other mutations may also interfere with the function of STK19 to bind DNA in the nucleus (Fig. 4a, colored orange). The latter could be categorized as follows: (1) Mutations of the basic residues that constitute the positively charged surface patch, including R192C, R216W, R205L, and R222L (Fig. 3a); (2) Mutations of the buried residues of the WH3-H1 helix, including F188V and G191E, which could destabilize this DNA binding helix; (3) Mutations of residues within the H3 recognition helix and the W1 wing of the WH2 and WH3 domains, for instance, R72M (WH1-H3), A75V (WH1-H3), R169G/L (WH2-W1), and G172V (WH2-W1); (4) Splice mutations and truncating mutations that alter the protein-coding sequence and structural integrity; (5) Mutations of the predicted classical nuclear localization signal with a consensus motif (K[K/R]X[K/R], X: any residue)^31^, which may affect STK19 localization in the nucleus, namely K16N and R17M. Taken together, this implies that a considerable amount of somatic mutations found in cancer patients could interfere with the function of STK19 to bind DNA in the nucleus, and these patients could have defects in the transcription-coupled nucleotide excision repair.

## Materials and methods

### Protein expression and purification

The human *STK19* gene was cloned from KYSE-410 cells by using standard reverse transcription and PCR technologies. KYSE-410 cell was preserved and provided by Professor Ziming Dong (Department of Pathophysiology, School of Basic Medical Sciences of Zhengzhou University). The identity of this cell line was validated by STR analysis. The sequence obtained matches the human *STK19* isoform G11-Y-short (UniProt accession number P49842-4, 254 amino acids). For expression, all constructs were cloned into the pGEX-6p-1 vector (GE Healthcare. Chicago, IL, USA), and all constructs were verified by sequencing (Sangon Biotech. Shanghai, China). The amino acid sequence of the linker is LEVLFQGPLGS, and cleavage by the PreScission protease occurs between the Q and GPLGS residues. Thus, residues GPLGS are appended to the N-terminus of the STK19 protein.

For structural studies, GST-tagged STK19 (residues 31-254) was expressed in the *E. coli* strain BL21-CodonPlus (DE3). Cells were cultured in Luria–Bertani (LB) medium with 100 µg/ml ampicillin at 37 °C until the OD_600_ of the culture reached 0.8–1.0. Protein expression was induced by 0.25 mM isopropyl-β-D-thiogalactopyranoside (IPTG, Solarbio. Beijing, China) for 20 h at 16 °C. The cells were harvested by centrifugation at 4000 rpm (JLA-8.1 Rotor, Avanti J-26S XP Centrifuge). The harvested cell pellet was resuspended in lysis buffer (20 mM Tris–HCl, pH 8.0, 200 mM NaCl, and 10 mM dithiothreitol (DTT)) and disrupted by sonication. The lysates were cleared by centrifugation at 16,000 rpm (JA-25.5 Rotor, Avanti J-26S XP Centrifuge) for 30 min and applied to glutathione Sepharose 4B resin (GE healthcare). After extensive washing with lysis buffer, the beads were collected into a 10 ml column. On-column cleavage of the GST-tag was performed by the addition of homemade PreScission protease and gentle rotation at 4 °C overnight. The cleavage buffer consisted of 20 mM Tris–HCl, pH 8.0, 100 mM NaCl, and 10 mM DTT. The target proteins were eluted using the cleavage buffer, concentrated, and loaded onto an anion exchange HiTrap Q HP column (GE Healthcare). STK19 proteins were eluted with a linear NaCl gradient and further purified using a Superdex 200 Increase 10/300 gel filtration column (GE Healthcare) in buffer containing 20 mM Tris–HCl, pH 8.0, 150 mM NaCl, and 10 mM DTT. Purified proteins were flash-frozen in liquid nitrogen and stored at − 80 °C. Mutant proteins were purified similarly.

### Protein crystallization and structure determination

The purified STK19 protein was concentrated to 10 mg/ml, and subjected to crystallization screens by the sitting-drop vapor diffusion method at 16 °C. To set up crystallization trials, the protein was mixed with precipitant at a ratio of 1:1 using the mosquito crystallography robot (SPT Labtech. Cambridgeshire, United Kingdom). Multiple commercial kits were screened, including those from Hampton Research (Aliso Viejo, CA, USA) and Jena Bioscience (Jena, Germany). To improve the quality of the crystals, C116S/C136S/C240S mutation was introduced to potentially prevent aggregation and improve the homogeneity^32^. This mutation does not affect the oligomerization status of the protein, as judged by the gel filtration profiles compared with the wild-type protein. The best crystals were grown in the reservoir condition of 0.1 M Bis–Tris, pH 6.3, 2 M ammonium sulfate. Crystals were transferred to cryo solutions containing 25% PEG400 before being flash-frozen in liquid nitrogen. X-ray diffraction data was collected at The Shanghai Synchrotron Radiation Facility (SSRF) beamline BL17B1 at the wavelength of 0.977 Å. Data were processed with the XDS software (Version June 30, 2023; https://xds.mr.mpg.de/)^33^. The human STK19 structure was solved by the molecular replacement method (Phaser Version 2.8.3) using the AlphaFold model^34,35^. Manual model building was performed using Coot (Version 0.9.8.8)^36^ to improve the structure. The structure was refined with Phenix refine (Version 1.18.2-3874)^37^, and the final 1.32 Å STK19 structure has an *R*_work_ and *R*_free_ of 0.176 and 0.196, respectively. Data scaling, refinement, and validation statistics are listed in Table 1.

**Table 1:** Data collection and refinement statistics (molecular replacement).

Crystal	STK19-31-254
Data collection	
Space group	P 21 21 21
Cell dimension	
*a,b,c*(Å)	55.322, 97.836, 98.657
*α,β,γ*(°)	90, 90, 90
Resolution (Å)	49.33-1.32(1.34-1.32)^a^
*R*_merge_ (%)	6.0(136.8)
*I/σI*	17.1(1.2)
Completeness (%)	99.8(97.4)
Redundancy	6.5(5.9)
Refinement	
Resolution (Å)	31.17-1.32
Total No. reflection	125249
*R*_work_/*R*_free_	0.1762/0.1955
r.m.s.d. bonds/angles (Å)	0.010/1.261
Protein/Ligand/Solvent atoms	3594/15/359
Protein/Ligand/Solvent B-factors (Å^2^)	18.86/28.41/25.87
Ramachandran plot statistics	
Most favorable	99.1%
Additionally allowed	0.9%
Disallowed	0.0%

^a^Values in parentheses are for highest-resolution shell.

### Electrophoretic mobility shift assays (EMSA)

5' IRDye® 700 labeled oligonucleotide dT30 (30 mer consecutive thymines), its complementary strand dA30, 5' IRDye® 700 labeled rU30 (30 mer consecutive uracils), and its complementary strand rA30 were purchased from Integrated DNA Technologies (IDT). For assays in Figs. 3d, 4d, and Supplementary Fig. S2, 59 bp dsDNA was used. The sequence of one strand is 5’- GAGCTGCCGAATTCTACCAGTGCCTTGCTAGGACATCTTTGCCCACCTGCAGGTTCACC-3'. The complementary strands were annealed to obtain dsDNA or dsRNA, and were stored at − 80 °C until used.

For EMSA, increasing concentrations of the STK19 protein were incubated with labeled nucleic acids in buffer (20 mM HEPES, pH 7.4, 100 mM NaCl, 1 mM MgCl2, 1 mg/ml bovine serum albumin, 2 mM DTT, and 5% glycerol). Concentrations of the nucleic acids and proteins used in each experiment are indicated in the corresponding figure. The binding reactions were kept on ice for 30 min in a 20 µl total volume. After incubation, loading dye (50% glycerol, 0.001% bromophenol blue, 0.001% xylene cyanol) was added and samples were loaded onto a pre-run native 5% polyacrylamide gel (acrylamide/bisacrylamide 29:1) in 1X TBE buffer. Gels were run at 6 V/cm for 1.0 h, and were visualized using the Odyssey CLx Infrared Imaging System (LI-COR Biotechnology. Lincoln, Nebraska, USA). Otherwise, gels were stained with GelRed, and imaged using the UVP GelDoc-It TS2 Imager. For quantification, band intensities were determined using the ImageJ program (NIH). The fraction of nucleic acids bound was calculated from the band intensities using the expression: 100%-(unbound/free species in the control lane). The fraction bound was plotted versus the protein concentration, and fitted by non-linear regression assuming one site-specific binding model using the GraphPad Prism software (Version 8.0.2; https://www.graphpad.com/updates/prism-802-release-notes). The underlying equation is Fraction bound = Bmax*X/(Kd + X), where X is the protein concentration. Original and uncropped gels are presented in Supplementary Fig. S4.

### Supplementary Information

Supplementary Information.

## Data Availability

The coordinates and structure factors for the human STK19 structure was deposited in the Protein Data Bank under accession number: 8YCM.
