# Supplementary material for: Mutations found in cancer patients compromise DNA binding of the winged helix protein STK19
Source: Sci Rep. 2024 Jun 18;14:14098. doi: 10.1038/s41598-024-64840-9 (PMC11189558; doi:10.1038/s41598-024-64840-9)
Supplement: Supplementary file 1 — Supplementary Information. [file 41598_2024_64840_MOESM1_ESM.pdf]

Mutations found in cancer patients compromise DNA binding of the winged helix protein STK19

Jian Li[1,†], Xinli Ma[1,†], Xiaoyu Wang[1,2,†], Xiaotong Hu[1,2,†], Shaobo Fang[1], Guoguo Jin[1,3], Kangdong Liu[1,2], Zigang Dong[1,2,*]

[1] China-US (Henan) Hormel Cancer Institute, Zhengzhou, Henan, 450003, China

[2] Department of Pathophysiology, School of Basic Medical Sciences, Zhengzhou University, Zhengzhou, Henan, 450001, China.

[3] Henan Key Laboratory of Chronic Disease Management, Fuwai Central China Cardiovascular Hospital, Zhengzhou, Henan, 450000, China

* To whom correspondence may be addressed. Email: dongzg@zzu.edu.cn

† These authors contributed equally.

Supplementary Figure S1

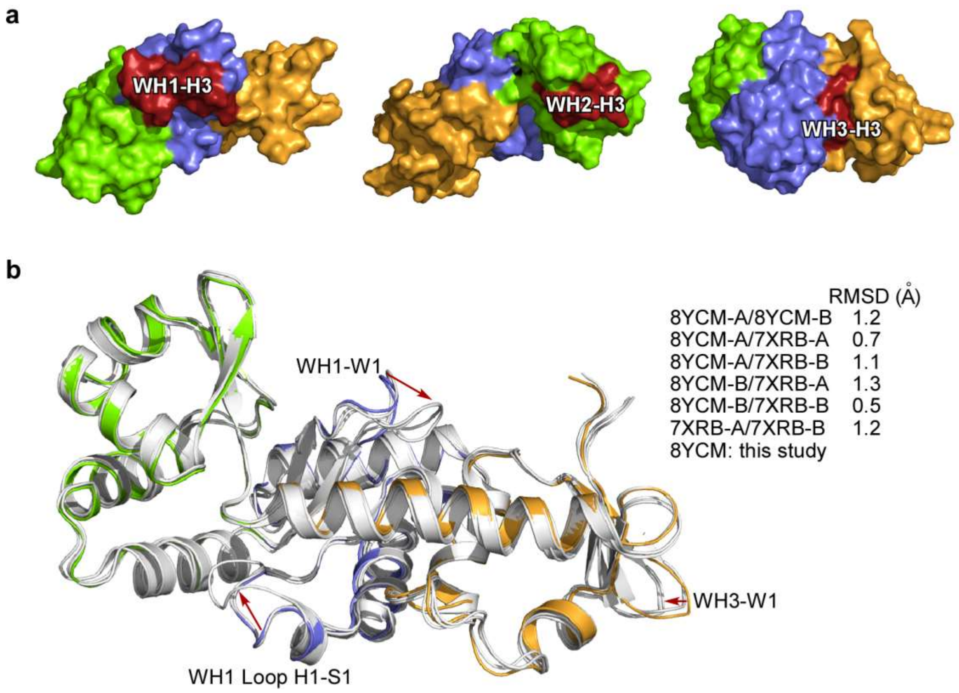

Supplementary Figure S1. Structural analysis of the human STK19 protein. (a) Surface representations showing the area of the H3 nucleic acid recognition helix. Compared to the WH1-H3 and WH2-H3, the WH3-H3 is less accessible, owing to its proximity to WH1-H2. The three WH domains are colored as in Fig. 1a, and the helices H3 are colored red. (b) Alignment of different chains of the STK19 structures (PDB ID 8YCM, 7XRB) reveals rigid and flexible portions of the protein. The overall structure is quite similar, as reflected by the low root-mean-square deviation (RMSD) of aligned C-alpha atoms. Meanwhile, three loops, the WH1-W1, WH3-W1, and the loop between H1-S1 in the WH1 domain are able to adopt different conformations (red arrow).

Supplementary Figure S2

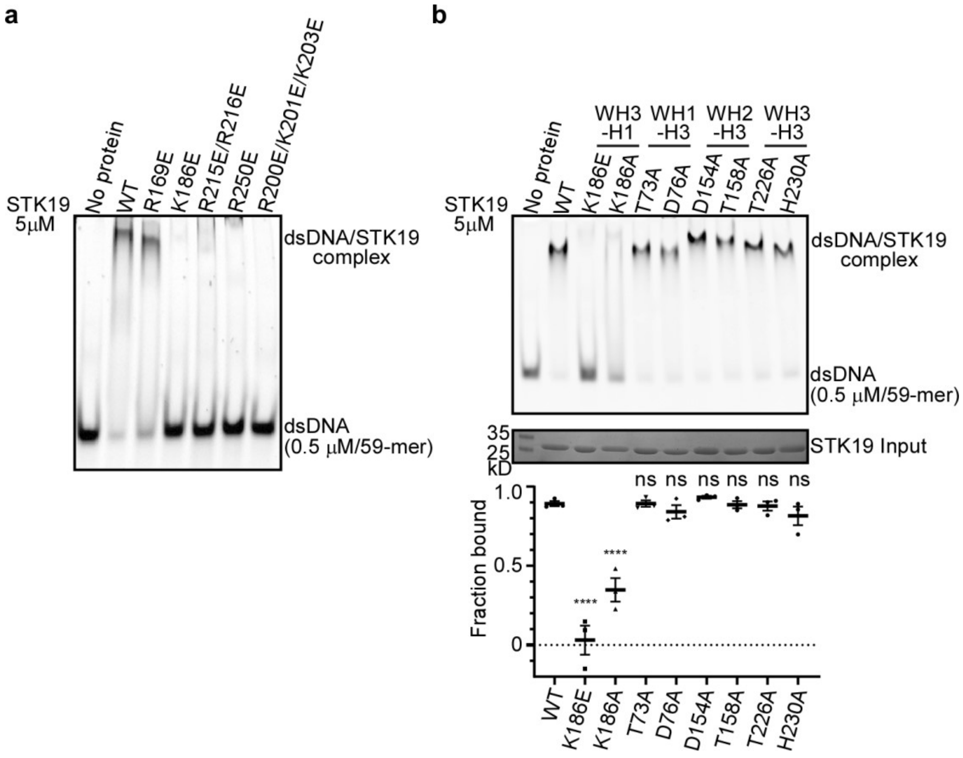

Supplementary Figure S2. Mutations that affect STK19 dsDNA binding. (a) Initial screen of a panel of STK19 mutants for their dsDNA binding ability. A 59-bp dsDNA was used. Representative images of EMSA are shown. (b) Conserved residues from the WH domain H3 recognition helices, T73, D76, D154, T158, T226, and H230, do not contribute to dsDNA binding. One-way ANOVA with Tukey's multiple comparisons test was used to evaluate the statistical significance. ****, $P < 0.0001$; ns, not significant. The significance indicated is in comparison to the WT protein. A 59-bp dsDNA was used, and representative images of EMSA are shown.

Supplementary Figure S3

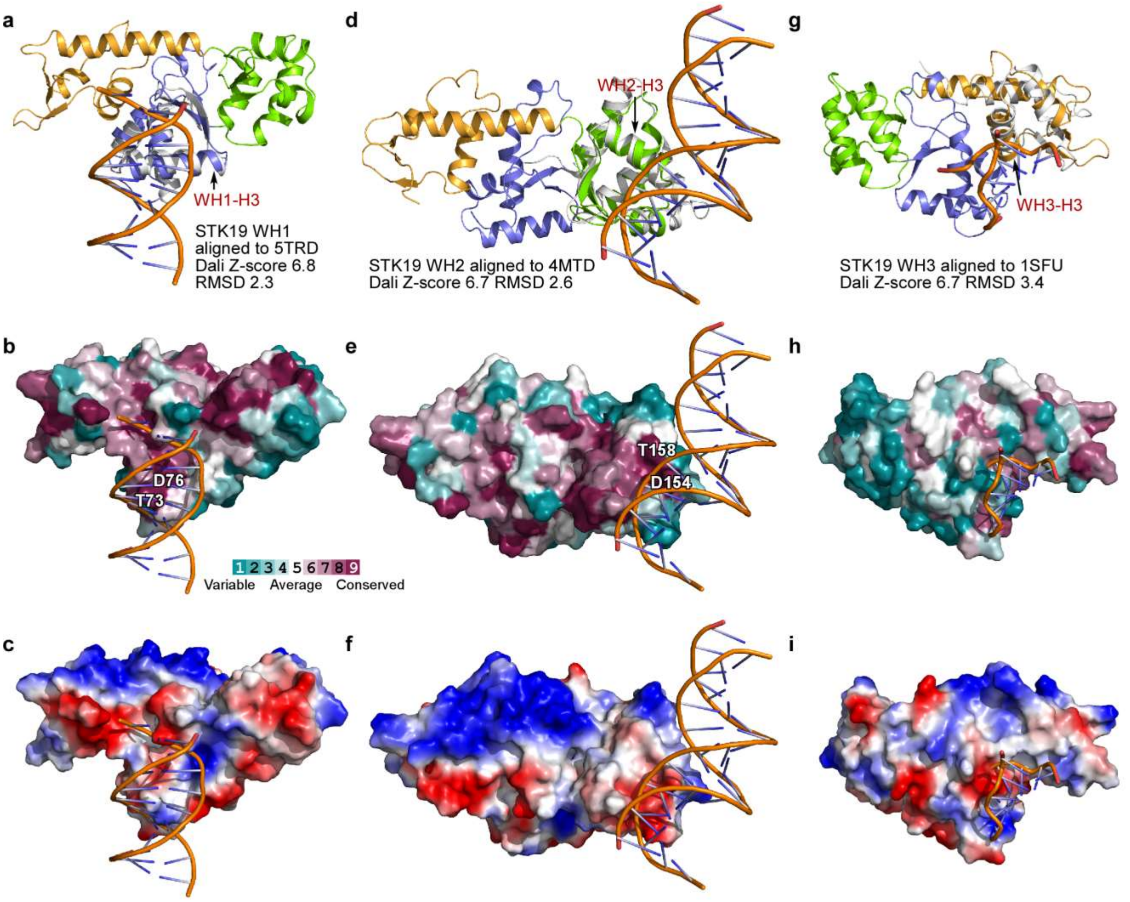

Supplementary Figure S3. Comparison of each of the STK19 WH domains to top hit identified by the DALI server. (a) Alignment of the STK19 WH1 to the structure of riboflavin kinase in complex with its cognate DNA operator (PDB ID 5TRD, chain A, colored gray). The STK19 WH1 may be compatible with dsDNA binding. WH domains are color-coded as in Fig. 1a, and the recognition helices are indicated by black arrows. (b, c) Surface conservation and charge distribution of the same view in (a). Residues T73 and D76 of the STK19 WH1 recognition helix are extremely conserved. (d) Alignment of the STK19 WH2 to the structure of zinc uptake regulator complexed with the cognate promoter (PDB ID 4MTD, chain D, colored gray). The STK19 WH2 may be compatible

with dsDNA binding. (e, f) Surface conservation and charge distribution of the same view in (d). Residues D154 and T158 of the STK19 WH2 recognition helix are highly conserved. (g) Alignment of the STK19 WH3 to the structure of the viral Zalpha domain bound to left-handed Z-DNA (PDB ID 1SFU, chain A, colored gray). The STK19 WH3 may not be optimal for canonical dsDNA binding, due to steric clashes between the adjacent WH1 domain and the modeled DNA. (h, i) Surface conservation and charge distribution of the same view in (g).

Supplementary Figure S4

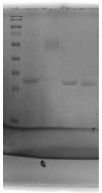

Fig. 1e

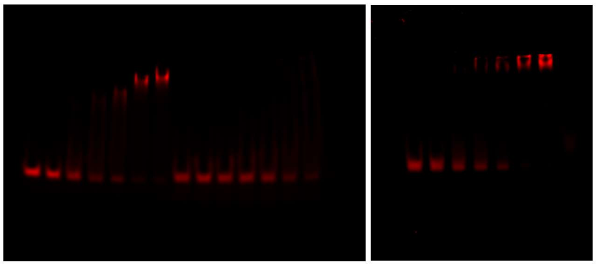

Fig. 2a

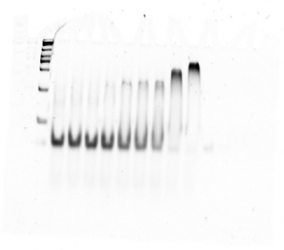

Fig. 3d WT

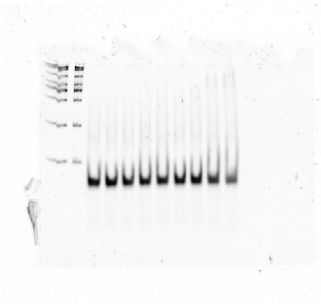

Fig. 3d K186E

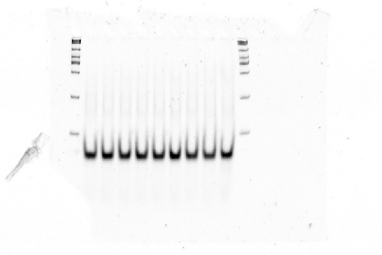

Fig. 3d R200E/K201E/K203E

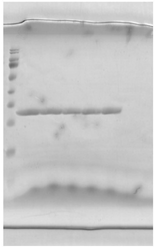

Fig. 4c

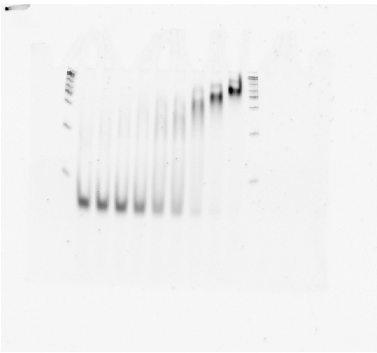

Fig. 4d WT

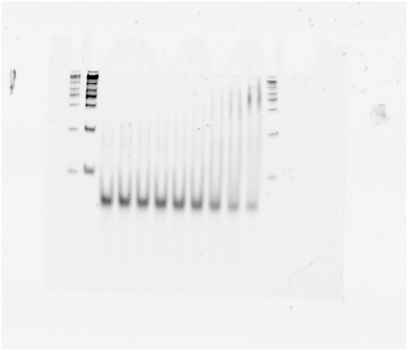

Fig. 4d K186N

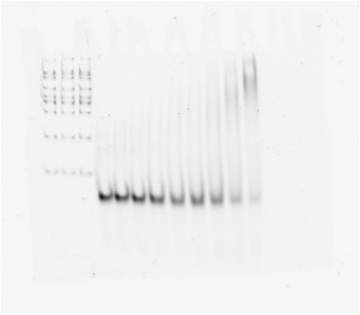

Fig. 4d R200W

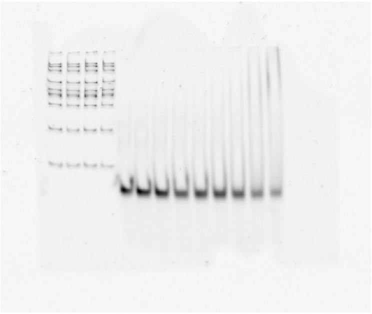

Fig. 4d R215W

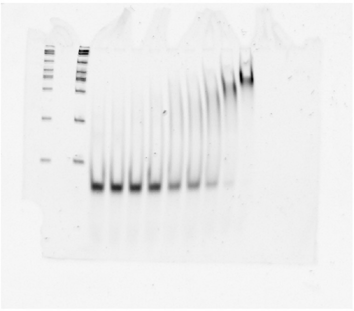

Fig. 4d A221V

Supplementary Figure S4. Original and uncropped gels.
